# Modelling the effects of intervention measures in reducing the risk of norovirus transmission in preschool settings

**DOI:** 10.1371/journal.pone.0314586

**Published:** 2024-12-03

**Authors:** Edward Lee, Benjamin Er, Joanna Khoo, Sheot Harn Chan, Kyaw Thu Aung

**Affiliations:** 1 National Centre for Food Science, Singapore Food Agency, Singapore, Singapore; 2 Department of Food Science and Technology, National University of Singapore, Singapore, Singapore; 3 School of Biological Sciences, Nanyang Technological University, Singapore, Singapore; Universidade de Lisboa Instituto Superior Tecnico, PORTUGAL

## Abstract

Singapore has seen an increase in norovirus outbreaks in preschools from 2019 to 2022 primarily due to person-to-person transmission. This study describes the use of compartmental susceptible-exposed-infectious-recovered (SEIR) modelling to simulate the spread of norovirus among preschool children in a classroom setting. Different intervention measures, such as isolation of infected preschoolers, handwashing and surface decontamination were modelled to quantify their effectiveness at reducing the number of children infected. We found that isolation of the sick child was the most effective single intervention to reduce transmission risk, which was 5 times more effective than handwashing. Coupled with handwashing and surface decontamination, transmission risk could be further reduced, close to zero. Findings from our study can be used to reiterate to parents and teachers on the importance of recognizing the symptoms exhibited by their unwell children and refraining from sending them to school, as this action poses a risk of transmitting norovirus to other children. In addition, our findings can be used to educate children and staff in preschools on the importance of practising good personal hygiene and regular environmental cleaning. Through this study, decision makers would be better informed on the effectivness of various risk mitigation measures at reduce the risk of norovirus transmission in preschool settings.

## Introduction

Norovirus is a highly contagious virus that causes viral gastroenteritis, commonly known as the stomach flu. It is characterized by symptoms such as vomiting, diarrhea, nausea, and stomach cramps. It can spread through person to person contact, consumption of contaminated food, or by touching contaminated surfaces. Subsequently, the virus can infect an individual when it enters the body via the mucosa membrane through contact with the nose, mouth or eyes [1]. Symptoms usually appear within 12 to 48 hours of exposure to the virus and can last for 1 to 3 days [2]. Norovirus outbreaks often occur in places where people are in close proximity [3], such as schools, military camps and cruise ships. Singapore saw an increase in the number norovirus outbreaks in preschools from 2019 to 2022 that were primarily of non-foodborne transmission, due to the lack of compliance to proper disinfection regimes [4]. Intervention measures aimed at reducing the risk of transmission include regular hand washing, surface decontamination, and isolation of individuals infected with norovirus; but the magnitude to which these measures reduce transmission is not well studied in preschool settings. S. Towers et. al. conducted a study to quantify the relative impact of environmental and direct transmission of norovirus on a cruise ship [5]. However, this may not be directly applicable to other settings like preschools.

Compartmental modeling in epidemiology is a method to simulate number of cases resulting from an infectious disease outbreak and has been used to succesfully to model Covid-19 transmission [6]. It can also be used used to quantify the impact of interventions on the spread of an infectious disease through the population over time, through a compartmental susceptible-exposed-infectious-removed (SEIR) model. This is a suitable approach for modeling the transmission of a viruses because it allows the representation of different stages of infection and the movement of individuals between these stages. The model divides the population into different compartments based on their disease status, such as susceptible, exposed, infected, and recovered. It also facilitates the evaluation of the impact of different interventions on the spread of the disease. By tracking the movement of individuals between these compartments, insights into the transmission dynamics of the disease and the effectiveness of different interventions could be identified. This makes it a powerful tool for understanding the transmission of viruses and designing effective public health interventions.

The purpose of our study is to understand how different interventions targeting the risk factors for norovirus outbreaks in preschools can impact the number of cases resulting from such an outbreak. Due to the lack of knowledge in the transmission of norovirus in preschool settings, we embarked on this study to simulate and identify effective intervention measures which can reduce the risk of norovirus transmission. The simulated output using transmission dynamics could be useful in informing public health and relevant authorities to identify key risks and prioritize disease prevention guidelines to preschools for compliance.

## Methods

### Transmission pathway of norovirus

In this study, the SEIR model was used [7] and additional compartments were added to handle the transmission of norovirus between individuals and commonly-touched surfaces. The model was adopted from Kraay et al [8] with additional compartments included based on the characteristics of the virus (i.e. norovirus can remain viable on fomites) and the transmission setting (i.e preschools).

### Model compartments

**Fig 1:**
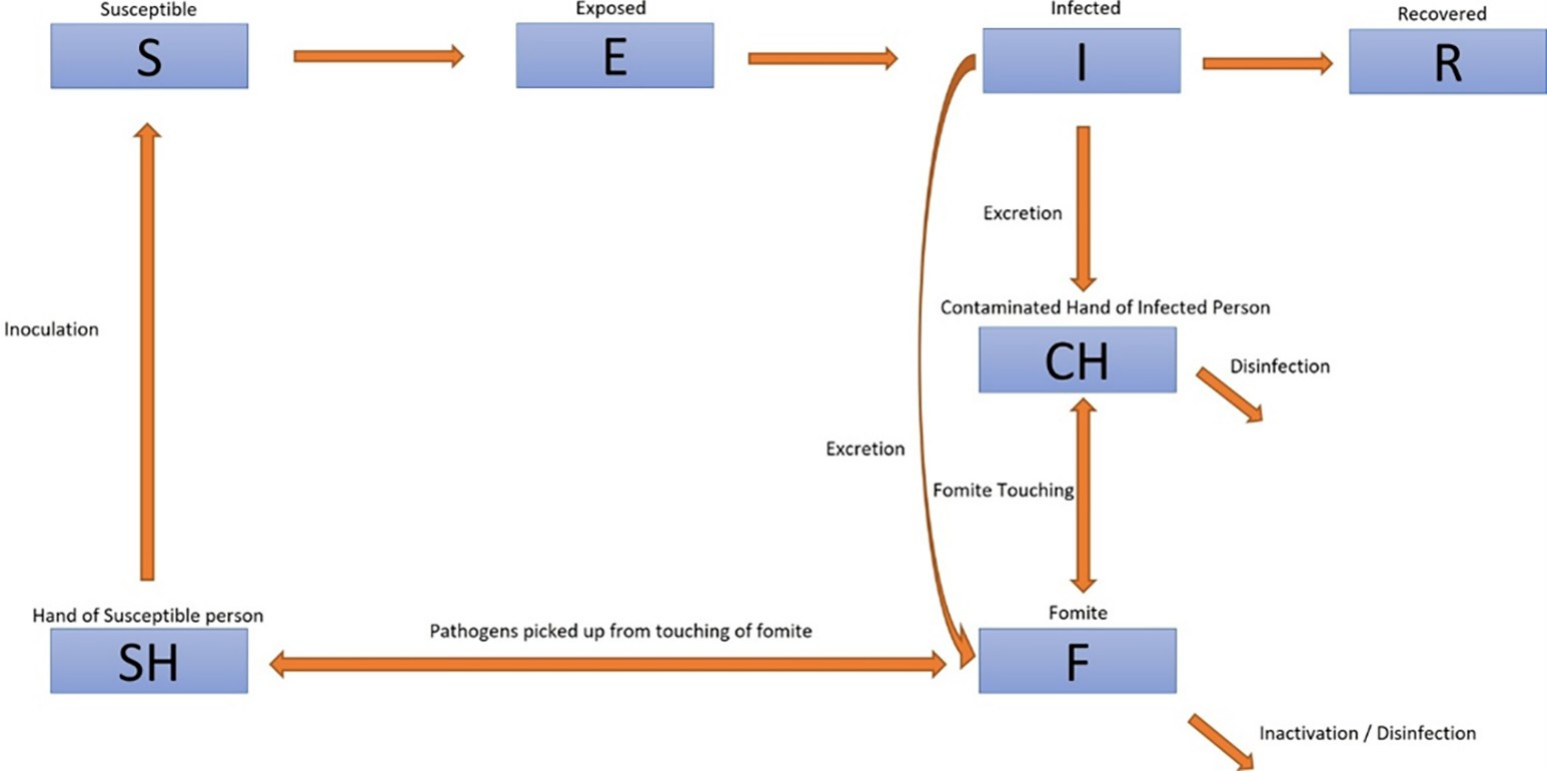
Transmission flow within compartmental model.

To apply the SEIR model, we first established a hypothetical scenario of the modes of transmission and transmission pathways based on our understanding of the spread of norovirus in a preschool classroom setting, and modelled them based on compartmental modelling techniques. We assumed that virus transmission could occur if an infected preschool child deposits viral particles onto contact surfaces through coughing, sneezing or by touching surfaces with his or her contaminated hands. These viral particles could subsequently be picked up by susceptible preschoolers when they come into contact with these contaminated surfaces, i.e. fomites. A susceptible preschooler could then get infected with norovirus if the viral particles enter their body when they rub their eyes or put fingers into their mouths. Fig 1 shows the different compartments in the model and what each compartment represents is further elaborated in the following section.

1. “Compartment S” represents the initial susceptible population of preschoolers. This group of children has not been exposed to or infected with norovirus.
2. “Compartment E” represents preschoolers that have been exposed to the virus and are asymptomatic. This group of preschoolers will remain in this compartment during the incubation period of the virus.
3. “Compartment I” represents preschoolers who are infected after the incubation period of the virus has ended.
4. “Compartment R” represents preschoolers who have recovered from the infection, and recovery rate was used to calculate the population that has recovered from the infection.
5. “Compartment CH” represents the hands of infected preschoolers. This compartment contains the pathogen count on preschoolers’ hands, where pathogens are deposited through coughing and sneezing, etc.
6. “Compartment F” represents fomites which are commonly-touched surfaces like tables, chairs and doorknobs, etc.
7. “Compartment SH” represents the hands of susceptible preschoolers. They have not been contaminated with the virus yet, however, they can pick up the virus by touching contaminated surfaces, resulting in their hands being contaminated with the virus.

### Modelling the flow of transmission between compartments

**Fig 2:**
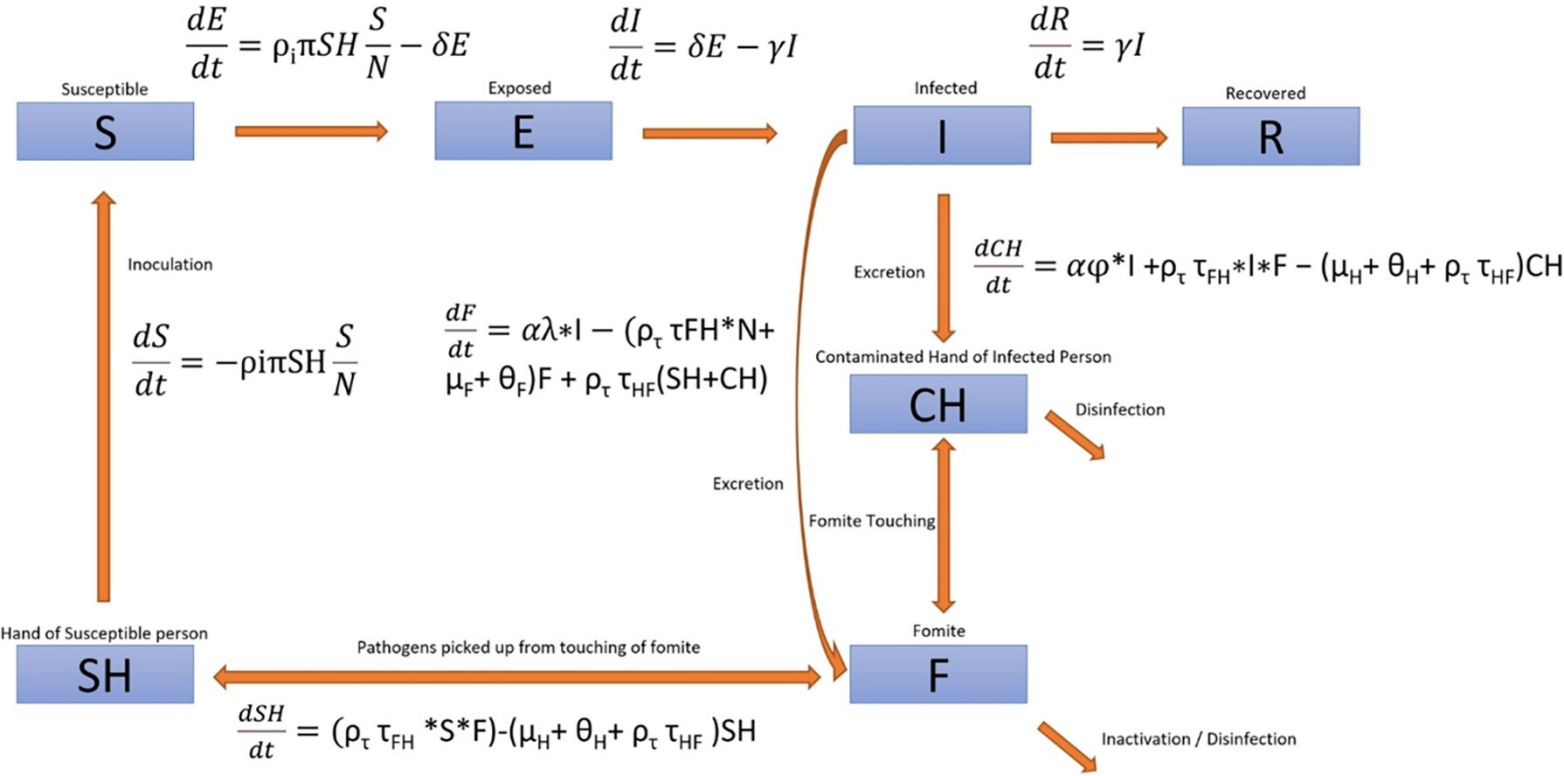
ODEs simulating the flow between each compartment.

The follow Ordinary Differential Equations (ODE) were used to model the flow between compartments. The ODE describing the transmission dynamics are described in Fig 2 below:

$$1)\;\frac{dS}{dt}=-\rho_{i}\pi SH\frac{S}{N}$$

This equation calculates the rate of change of susceptible preschoolers that will be exposed to the virus (asymptomatic). It takes the product of the infectivity rate (π), inoculation rate (ρ_*i*_), pathogen count on the hands of susceptible preschoolers (SH) and the proportion of preschoolers that are susceptible. Inoculation rate refers to the frequency at which a child’s hands come into contact with his/her eyes or mouth.


$$2)\;\frac{dE}{dt}=\rho_{i}\pi SH\frac{S}{N}-\delta E$$

This equation takes in the output from the first equation which equates to the number of susceptible preschoolers who were exposed to the virus per unit time ($\rho \pi SH\frac{S}{N}$). The rate of change in the number of preschoolers who turned symptomatic after the incubation period of the virus (*δE*) were subtracted from this. An incubation period of 2 days was assumed in the model.


$$3)\;\frac{dI}{dt}=\delta E-\gamma I$$

This equation calculates the rate of change in the number of exposed preschoolers who turned symptomatic (*δE*) and the number of infected preschoolers who recovered (*γI*). This takes into consideration the recovery rate (*γ*) of preschoolers. In the model, preschoolers were assumed to have a recovery period of 10 days.


$$4)\;\frac{dR}{dt}=\gamma I$$

This equation describes the flow of preschoolers from the infected to the recovered compartments. It calculates the rate of change in the number of infected preschoolers who recovered.


$$5)\;\frac{dCH}{dt}=\;\alpha \varphi I\;+\;\rho_{T}\tau_{FH}\;IF\;-(\mu_{H}+\;\theta_{H}+\rho_{T}\tau_{HF})CH$$

This equation calculates the rate of change in the pathogen count on the hands of preschooler infected the virus. Viral particles can be deposited onto the hands of an infected preschooler through coughing, sneezing, or droplets produced from talking. This is calculated by taking the product of the viral shedding rate (*α*), proportion of pathogens deposited onto the hands of an infected preschooler (*φ*) and the number of infected preschoolers (*I*). The count of pathogens picked up from fomites onto the hands of infected preschoolers was calculated by taking the product of the frequency of contact with fomites (*ρ*_*T*_), transfer efficacy from fomites to hands (*τ*_*FH*_), the number of susceptible preschoolers (*I*) and the pathogen count on fomites (*F*). Reduction of pathogen count on hands (*CH*) was calculated by subtracting the product of the rate of natural decay of the virus (*μ*_*H*_), frequency of handwashing (*θ*_*H*_), frequency of contact with fomite (*ρ*_*T*_) and the transfer efficacy of virus from hand to fomite (*τ*_*HF*_).


$$6)\;\frac{dF}{dt}=\alpha \lambda \;I-(\rho_{T}\tau_{FH}\;N+\;\mu_{F}+\;\theta_{F})F\;+\;\rho_{\tau }\tau_{HF}(SH+CH)\;$$

This equation calculates the rate of change in the pathogen count deposited onto fomites. Pathogens can be deposited directly onto fomites when an infected preschooler coughs or sneezes onto surfaces. This is calculated by taking the product of the viral shedding rate (*α*), proportion of pathogens deposited onto fomites (*λ*) and the number of infected preschoolers (*I*). Pathogens can also be deposited onto fomites from the hands of preschoolers that have been contaminated with the virus. This is calculated by taking the product of the frequency of contact with fomites (*ρ*_*T*_), transfer efficacy of virus from hand to fomite (*τ*_*HF*_) and the pathogen count on SH and CH. Pathogen count on fomites (*F*) is reduced through natural decay of the virus (*μ*_*F*_), surface decontamination (*θ*_*F*_) and preschoolers picking up the virus from fomites, which is calculated by taking the product of the frequency of contact with fomites (*ρ*_*τ*_), the transfer efficacy of virus from fomite to hand (*τ*_*FH*_) and the total population in the classroom (*N*).


$$7)\;\frac{dSH}{dt}=\;\rho_{\tau }\tau_{FH}SF-(\mu_{H}+\;\theta_{H}+\rho_{\tau }\tau_{HF})SH$$

This equation calculates the rate of change in the pathogen count on the hands of susceptible preschoolers. Preschoolers can pick up pathogens from fomites through random contact with these surfaces. This is calculated by taking the product of the frequency of contact with fomites (*ρ*_*τ*_), transfer efficacy from fomite to hand (*τ*_*FH*_), the number of susceptible preschoolers (*S*) and the pathogen count on fomites (*F*). Pathogen count on hands (*SH*) is reduced through natural decay of the virus (*μ*_*H*_), handwashing (*θ*_*H*_) and preschoolers depositing the virus from their hands onto fomites, which is calculated by taking the product of the frequency of contact with fomites and the transfer (*ρ*_*τ*_*τ*_*HF*_).

### Parameter values

Parameters used to model the flow between compartments, and the full list of parameter values referenced from research papers [8–11] are shown in Table 1. The parameter values for viral infectivity rate (π), inoculation rate (ρ_*i*_), shedding rate (*α*), transfer efficacy from fomite to hand (*τ*_*FH*_), transfer efficacy from hand to fomite (*τ*_*FH*_), inactivation rate (*μ*_*H*_) and inactivation rate on fomite (*μ*_*F*_) used in our paper were referenced from a meta-analysis of fomite-mediated transmission of norovirus conducted by Kraay et al [8]. From the same paper, the parameter values representing the proportion of viral particles that get deposited onto hands (φ) and contact surfaces (λ) were adjusted from 0.9 to 0.5 and from 0.1 to 0.5 respectively to simulate the more likely scenario where a sick child coughs or sneezes and viral particles are emitted into the environment and deposited indirectly onto hands or contact surfaces with equal probability. The parameter values representing the rate of handwashing (*θ*_*H*_) and surface decontamination (*θ*_*F*_) were assumed to be once per hour under the baseline simulation scenario. Lastly, the parameter value representing the rate of fomite contact (*ρ*_*τ*_) was normalised from 60 times per day to 10 times per hour assuming 6 hours in the classroom. The parameter values representing the virus incubation period in days (*δ*) and the recovery period in days (*γ*) were referenced from [1]. Cases typically recover from norovirus infection within 1 to 3 days; however, the virus may continue to be shed for several weeks. Therefore, the recovery period in the simulation was extended to 10 days to adopt a more conservative approach.

**Table 1:** Model parameter values.

	Parameter	Value	References
1	π—Infectivity rate	4.78e-4	[8]
2	*ρ*_*i*_—Inoculation rate per hour	15.8	[8]
3	*δ*—Incubation period (*days*^−1^)	2	[1]
4	*γ*—Recovery period (*days*^−1^)	10	[1] with adjustment
5	*α*—Shedding rate per hour	2.88 *x* 10^3^	[8]
6	*φ*—Proportion of pathogen excreted to hand	0.5	[8] with adjustment
7	*ρ*_*T*_—Rate of fomite contact per hour	10	[8] with adjustment
8	*τ*_*FH*_—transfer efficacy of pathogen from fomite to hand per hour	0.07	[8]
9	*τ*_*HF*_—transfer efficacy of pathogen from hand to fomite	0.13	[8]
10	*μ*_*H*_–Inactivation rate of pathogen in hands per hour	1.07	[8]
11	*θ*_*H*_–Rate of handwashing per hour	1	Assumed
12	*λ*: Proportion of virus that settle on fomites	0.5	[8] with adjustment
13	*μ*_*F*_–Inactivation rate of pathogen on fomite per hour	0.288	[8]
14	*θ*_*F*_–Rate of fomite decontamination per hour	1	Assumed

### Modelling the impact of intervention measures

Handwashing, surface decontamination, and isolation of infected individuals were selected as intervention measures to be modelled because studies have iterated the importance of these measures at reducing the transmission of infectious diseases [8]. The impact of these 3 intervention measures were compared based on the resulting number of infected preschoolers at the end of the simulation runs.

Sensitivity analysis was carried out to examine how a delay in isolating an infected preschooler, frequency of handwashing and surface decontamination affect the total number of infected cases.

### Simulating the transmission of norovirus

The transmission of norovirus in a preschool setting was simulated with 29 susceptible and 1 infected preschooler for a period of 12 timesteps, with seven different combinations of interventions described in the section below. An arbitrary number of one infected preschooler was used to reflect the starting point of an outbreak in the model. Each timestep represents one hour and the number of infected cases resulting from the different interventional scenarios were compared at the end of the 12-hour mark. The number of preschoolers enrolled in a class varies depending on the preschool and it typically ranges from 15 to 20 children in a classroom [12]. A conservative number of 30 children was chosen and simulated in a classroom setting without mixing of preschoolers from other classrooms. Handwashing and surface decontamination performed at a frequency of once an hour was assumed due to the different cleaning procedures and frequency practiced in different preschools.

### Simulating different combinations of interventions

Seven different combinations of interventions were simulated to assess their effective in reducing the number of infected cases:

1. No intervention
  Simulation was performed without any intervention to quantify the number of infected cases at the end of the 12-hour period. This meant that susceptible and infected preschoolers were simulated to be free to mingle with one another, and with no handwashing and surface decontamination performed.
2. Surface decontamination
  Simulation was performed with surface decontamination of commonly-touched surfaces hourly to quantify the number of infected cases at the end of the 12-hour period. Susceptible and infected preschoolers were free to mingle with no handwashing performed.
3. Handwashing
  Simulation was performed with handwashing of preschoolers once every hour to quantify the number of infected cases at the end of the 12-hour period. Susceptible and infected preschoolers were free to mingle with no surface decontamination performed.
4. Handwashing and surface decontamination
  Simulation was performed with both handwashing and surface decontamination performed. Susceptible and infected preschooler were free to mingle with one another.
5. Isolation of infected person without handwashing and surface decontamination
  Simulation was performed with isolation of infected preschooler from the other preschoolers within an hour of symptoms. Handwashing and surface decontamination were not included in the simulation. The number of viral particles deposited onto commonly touched surfaces was simulated based on a period of one hour.
6. Closure of school
  Closure of school was simulated with susceptible preschoolers being sent home while the infected preschooler was sent to a clinic within an hour.
7. Isolation of infected person with handwashing and surface decontamination
  Simulation was performed with isolation of the infected preschooler within an hour. Simulation included handwashing and surface decontamination once every hour.

### Sensitivity analysis

The effectiveness of each intervention measure combination was determined by calculating the percentage reduction in the number of infected cases from the total population of 30 preschoolers at the end of 12 hours. Sensitivity analysis was then performed to compare the impact of changing the frequency of handwashing and surface decontamination from once per hour to once every 12 hours on the number of infected cases. The impact of isolating the infected preschooler at different timepoints from when symptoms were first displayed, ranging from within the first hour to the twelfth hour, was also assessed through a sensitivity analysis. All simulations, analyses and visualizations were performed and created using Python (version 3.8.8).

## Results

**Fig 3:**
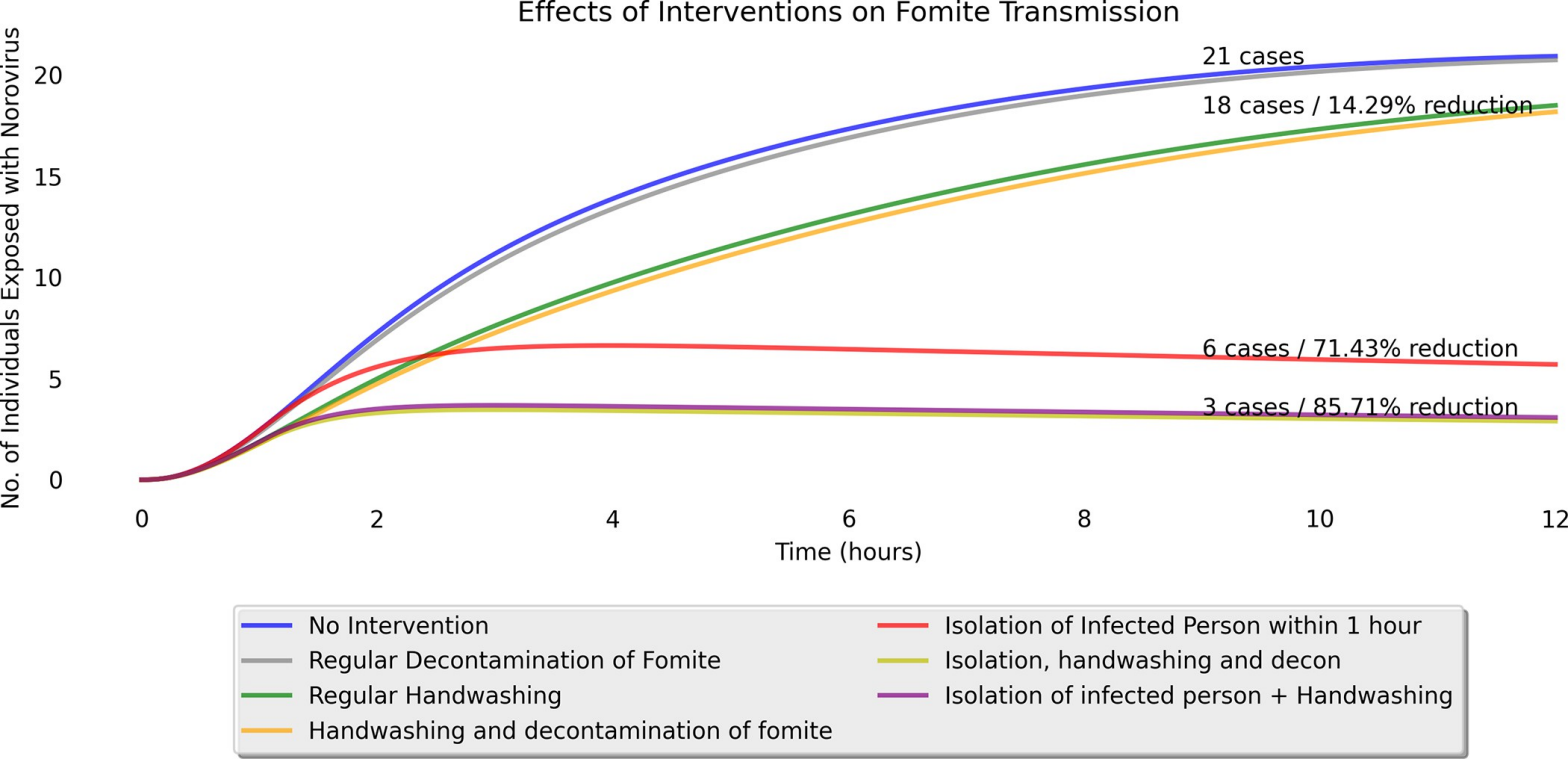
Simulation output for each intervention measure.

Fig 3 shows the results of the simulation under the seven intervention scenarios.

Without any intervention measure, the model estimated 21 infected cases out of 30 preschoolers at the end of the simulation as shown by the blue line in Fig 3. With intervention measures, surface decontamination (grey line), handwashing (green line) and both performed together (orange line) had no significant impact in relation to reduction in the number of infected cases. There were 20 infected cases for surface decontamination and 18 cases for both handwashing, and combined surface decontamination and handwashing interventions at the end of the simulation.

In contrast, isolation of the infected preschooler (red line) was shown to have a significant reduction in the number of infected cases at 6 cases (71.43% reduction), representing the single most effective intervention measure. Combining isolation, handwashing and surface decontamination together in a simulation achieved a 90.48% reduction in the number of infected cases, at 2 cases out of 30 preschoolers being infected, and highlights the synergistic effects of various intervention measures at reducing viral transmission.

**Fig 4:**
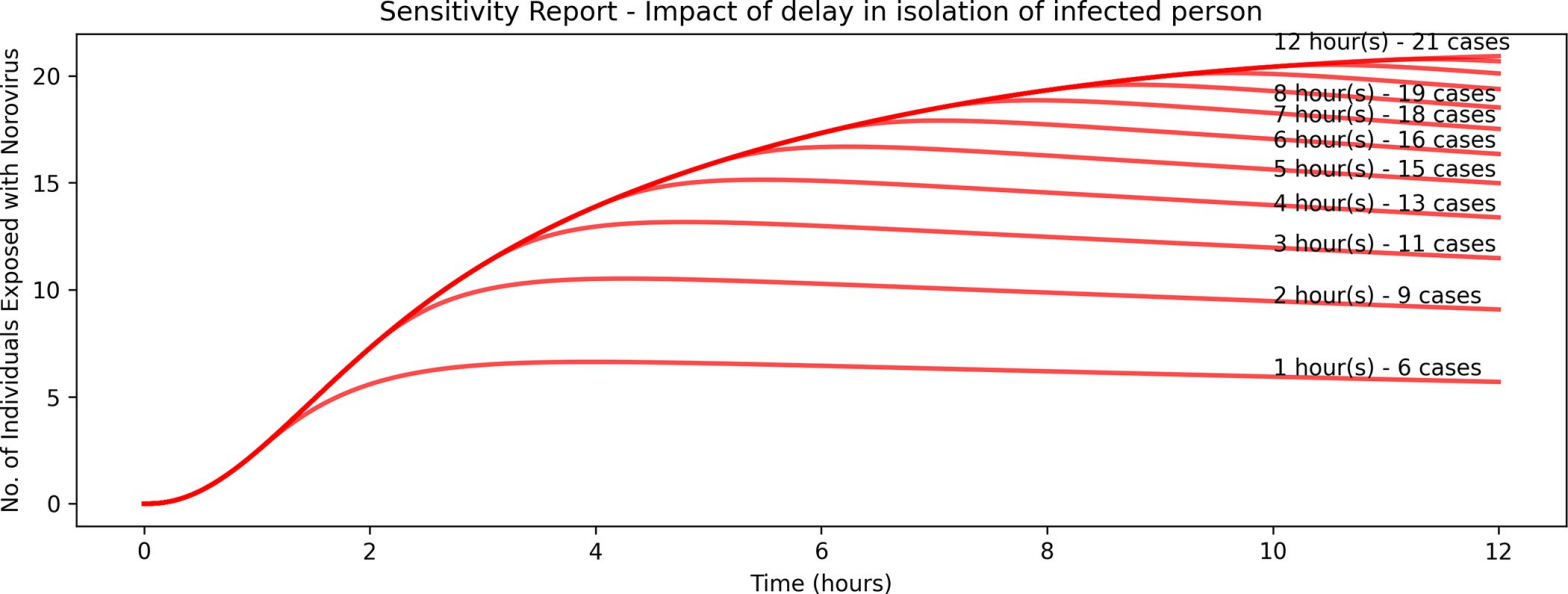
Sensitivity analysis of delay in isolation.

Fig 4 shows that the longer the delay in isolating infected preschoolers resulted in an increase in number of exposed cases for each passing hour. Isolating the infected preschooler within an hour resulted in 6 infected cases while delaying the isolation by another hour resulted in an additional 3 cases. The greatest impact was observed in the scenario where the infected preschoolers were isolated within one hour of being exposed to norovirus. Diminishing returns were observed from the third hour onwards. This is likely due to the reduced number of susceptible preschoolers remaining after every time step.

**Fig 5:**
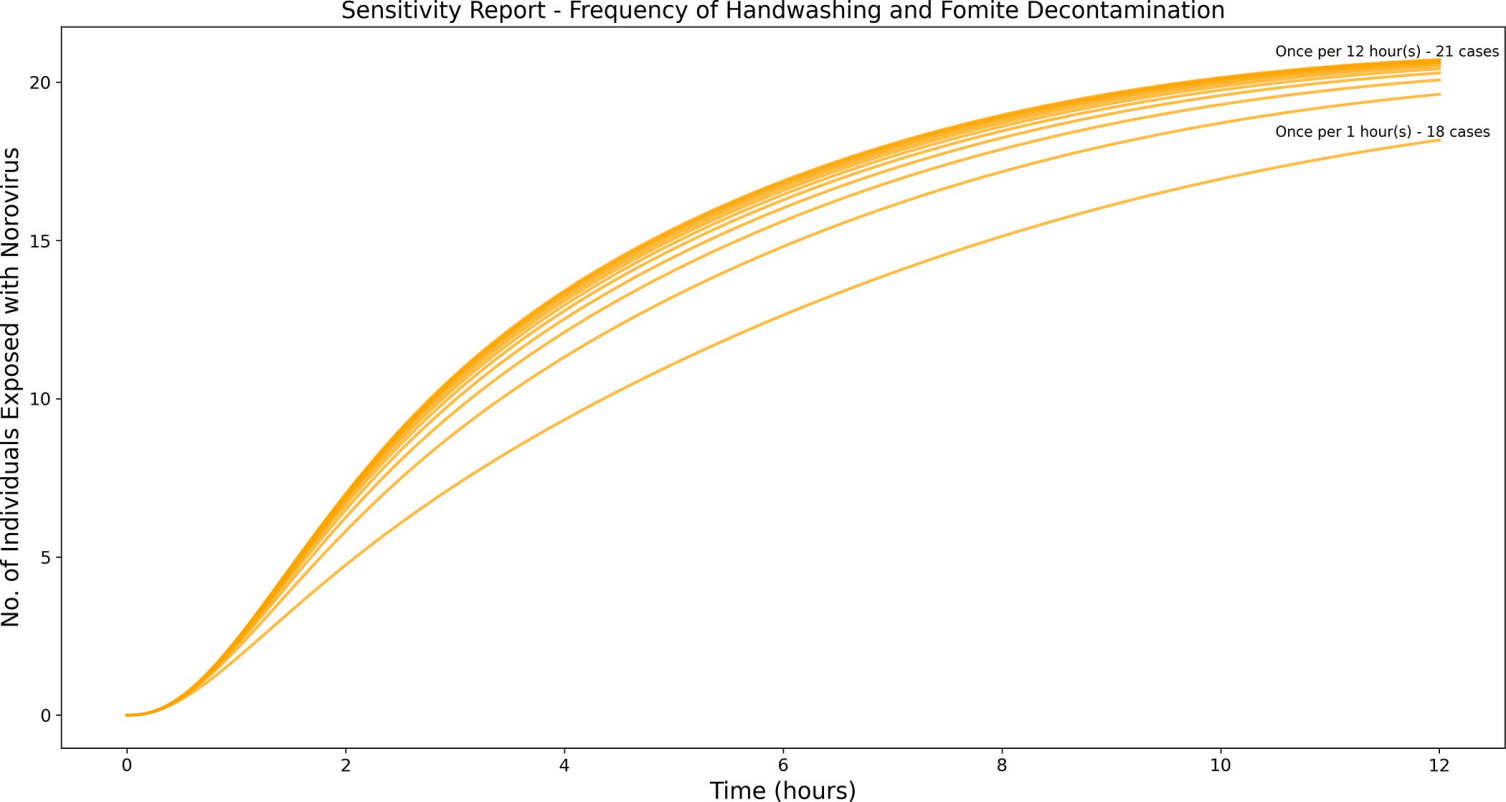
Sensitivity analysis of handwashing and surface decontamination.

Fig 5 shows that handwashing and surface decontamination alone, without isolation of the infected preschooler was not substantially effective at reducing transmission of norovirus and subsequent infection. Increasing the frequency of handwashing and surface decontamination from once every 12 hours to once every hour resulted only in a minor reduction in the number of infected cases.

## Discussion

This study aimed to elucidate and rank the most important risk factors for implementation of interventional risk control and management measures that are most impactful, and to support targeted communications plans in preschools. To our knowledge, this is the first study in Singapore that modelled the impact of intervention measures in reducing the risk of norovirus transmission in preschool settings.

The single most effective intervention measure was shown to be isolation of infected preschoolers to reduce exposure to norovirus. This observation has been corroborated by studies worldwide demonstrating the effectiveness of quarantine measures at preventing transmission of infectious diseases. For instance, Janneke C.M. Heijne et al found that strict isolation of patients were required to contain a norovirus outbreak [13]. In our study, isolation of the infected preschooler within an hour resulted in the number of infected cases peaking at the 2-hour mark. The likely reason is due to the removal of the transmission source and the weakening of the virus due to decay over time. However, isolating the infected preschooler within two hours instead of one increased the number of infected cases to 9, which suggests prolonged exposure of susceptible preschoolers to the transmission source (i.e. infected individual) with continued shedding of viral particles, increased their risk of infection. Diminishing returns were observed for isolations performed after the 3-hour mark, which is likely due to the reduced number of susceptible preschoolers who could be infected. In view of these reasons, it is paramount to remove the source of viral transmission as far as practicable through isolation. Anecdotal evidence suggests that parents tend to bring their sick child to school due to them perceiving certain gastroenteritis symptoms as mild and a lack of awareness of the transmissibility of such illnesses [14]. In addition, it may not be logistically feasible for parents to keep their sick child at home due to work commitments and a lack of an available caregiver. The key to such a social issue could be to strengthen caregiver support among families with young children. Advisories should serve as a reminder for parents not to bring their sick child to school in the first place rather than having to isolate them when symptoms start to show. It may also not be always easy to identify preschoolers infected with norovirus as gastroenteritis-like symptoms may also be a symptom of a multitude of other diseases. Preschool teachers could try to reduce the interactions between a sick preschooler and the rest of the class when symptoms start to show.

As isolation of the infected preschooler may not always be logistically feasible, handwashing and surface decontamination still play and important part in reducing the transmission of norovirus. There is a risk that susceptible preschool children come into contact with contaminated high-touch points and surfaces and getting infected with the virus upon rubbing their eyes, nose, or mouth with their hands. Thus, regular handwashing and surface decontamination would help to continually remove viral contaminants from the children’s hands and from the environment.

There are also other measures which can potentially reduce the transmission of norovirus such as reducing the sharing of soft toys amongst preschoolers, increasing the frequency of changing mob-heads for cleaning and increasing ventilation in classrooms. However, regular surface decontamination and handwashing are still important measures at reducing transmission risk through the elimination of direct virus transfer from surfaces onto hands and subsequently onto the mucosa membrane of susceptible individuals. Preschool management teams could step up outreach efforts to parents and communicate on the importance of joint responsibility of ensuring the personal hygiene of children, towards prevention of disease transmission in preschools.

The Environmental Infection Transmission Systems (EITS) model which was used in this paper was chosen over the Quantitative Microbial Risk Assessment (QMRA) model for modeling the spread of norovirus in a preschool setting due to its ability to capture the complexity and dynamics of infection transmission within specific environments. Preschools are intricate settings where norovirus can spread rapidly through various pathways, including person-to-person contact, contaminated surfaces, and shared objects. The EITS model is well-suited for simulating these multiple transmission routes and the interactions between them, providing a more holistic understanding of how norovirus spreads in such a dynamic environment.

The EITS model’s strength lies in its ability to incorporate detailed environmental factors and human behaviors that influence infection spread. In a preschool setting, factors such as the frequency and nature of interactions among children, hygiene practices, and cleaning routines play a crucial role in transmission dynamics. The EITS model can simulate these variables, allowing for a more accurate representation of real-world conditions. This level of detail is essential for identifying the most efficient intervention measures, as it enables the modeling of different scenarios and the evaluation of how changes in behavior, cleaning protocols, or environmental design can impact the spread of the virus.

In contrast, the Quantitative Microbial Risk Assessment (QMRA) model, while powerful in estimating risk from specific exposures, is more limited in its ability to account for the complex and interactive nature of multiple transmission routes within a confined space like a preschool. QMRA typically focuses on quantifying the risk associated with a single exposure route, making it less suitable for environments where multiple factors simultaneously contribute to the spread of infection. Therefore, the EITS model was selected for this study to provide a comprehensive and dynamic analysis of norovirus transmission and to inform targeted, context-specific interventions that can effectively reduce the risk of outbreaks in preschool settings.

Nonetheless, the models used in this study were entirely based on mathematical equations that describe the transmission of an infection disease in a populational setting. These models typically use statistical parameters to estimate the average behaviour of the population, and they assume that all individuals in the population behave in the same way. This is useful for predicting the overall behaviour of a system, but they may not capture individual-level interactions that drive the behaviour. In addition, the models are deterministic and simulations are based on a single classroom setting without the mixing of children from other classrooms. The use of stochastic parameter values with probability distributions to factor in randomness could more accurately reflect the real world. Other environmental and physical factors like ventilation, how preschoolers are grouped in classes and the size of classrooms were not studied. Effects of viral transmission through the sharing of soft toys and the impact of changing mob-heads frequently for cleaning were not modelled due to the lack of data. Validation of the model was also not possible due to insufficient empirical data from outbreaks, i.e. the number of preschoolers per class, frequency of handwashing and surface decontamination etc. Hence, the simulations might not be very realistic. More research needs to be done to holistically understand the impact of all these factors and to validate the results once more data becomes available in the future.

### Conclusion

Our study corroborates the knowledge that isolating an infected child is the single most effective intervention measure at preventing norovirus transmission in preschools, notwithstanding the fact that symptoms of the disease may not be easy to identify nor that it may not be logistically feasible for parents to care for their sick child at home. Other measures such as handwashing and surface decontamination would thus still play some role at reducing transmission as much as practicable in a preschool setting. Isolation together with handwashing and surface decontamination is the most effective combined intervention measure if isolation is logistically feasible to be performed.

Effective intervention measures identified in this study can potentially help to reduce the number of norovirus incidents in preschool environments through inculcation of good hygiene practices to teachers and parents of preschool children. The simulated results would be useful in helping decision makers prioritise disease prevention guidelines to preschools for compliance, though further studies are required to understand other intervention measures that may also be effective in complementing existing measures at preventing norovirus outbreaks in preschools.

## Supporting information

S1 File Supplementary SEIR norovirus-fomite transmission model. (TXT)
